# miRNAs Dysregulated in Human Papillomavirus-Associated Benign Prostatic Lesions and Prostate Cancer

**DOI:** 10.3390/cancers17010026

**Published:** 2024-12-25

**Authors:** Sandra Viridiana Salgado-Hernández, Lucero Martínez-Retamoza, Rodolfo Ocadiz-Delgado, Salvador Pérez-Mora, Gladys Edith Cedeño-Arboleda, María del Consuelo Gómez-García, Patricio Gariglio, David Guillermo Pérez-Ishiwara

**Affiliations:** 1 Laboratorio de Biomedicina Molecular I, Programas de Doctorado en Ciencias en Biotecnología y Maestría en Biomedicina Molecular, Escuela Nacional de Medicina y Homeopatía (ENMyH), Instituto Politécnico Nacional, Mexico City 07320, Mexico; ssalgadoh1600@alumno.ipn.mx (S.V.S.-H.); luceromartinezretamoza@gmail.com (L.M.-R.); sperezm1510@alumno.ipn.mx (S.P.-M.); gcedenoa1900@alumno.ipn.mx (G.E.C.-A.); cgomezg@ipn.mx (M.d.C.G.-G.); 2 Department of Genetics and Molecular Biology, Centro de Investigación y de Estudios Avanzados del IPN (Cinvestav), Mexico City 07360, Mexico; wilox@cinvestav.mx (R.O.-D.); vidal@cinvestav.mx (P.G.)

**Keywords:** miRNAs, HPV, prostate cancer, BPH, prostatitis, interactomic analysis, functional enrichment analysis, inflammation, proliferation, apoptosis, angiogenesis, metastasis

## Abstract

**Background/Objectives**: Chronic inflammation is a critical factor in the development of prostatic diseases, including prostatitis, benign prostatic hyperplasia (BPH), and prostate cancer (PCa). Although this is recognized as a significant risk factor, the molecular mechanisms underlying these conditions remain unclear. Studies have shown that human papillomavirus (HPV) can infect the prostatic epithelium; however, its role in prostatic pathologies is not fully understood. This study aimed to evaluate the expression profiles of miRNAs involved in inflammation, proliferation, cell cycle regulation, apoptosis, angiogenesis, migration, invasion, and metastasis in HPV-positive and HPV-negative prostatic tissues. **Methods**: Using bioinformatics analyses, including interactomic and functional enrichment analyses, miRNAs associated with PCa and their potential roles in key cellular processes related to tumor progression were identified. Subsequently, some of these miRNAs were selected to evaluate their expression profiles in biopsies from patients with BPH/prostatitis and PCa, including HPV-positive and HPV-negative samples. **Results**: Our results revealed that 67.2% of benign lesions and 93.4% of cancer samples were HPV-positive. Interactomic analysis identified 284 miRNAs associated with PCa, with key miRNAs such as let-7c, miR-21, miR-34a, miR-126, miR-18a, miR-145, miR-221, miR-106a, miR-222, and miR-143 highlighted. Dysregulated expression of miRNAs was observed in HPV-positive tissues: miR-34a, miR-143, and miR-145 were downregulated in BPH/prostatitis, while miR-221 was upregulated. In HPV-positive PCa, additional miRNAs, including let-7c, miR-126, and miR-106a, showed altered expression. Complementarily, the functional enrichment analysis revealed that these miRNAs are associated with key biological processes that are crucial to cancer, such as cell cycle regulation, apoptosis, immune response, angiogenesis, metastasis, and tumor suppressor pathways. This finding is consistent with the predicted target genes of the evaluated miRNAs and their potential roles in processes related to cancer development and progression. **Conclusions**: The differential expression of HPV-induced miRNAs suggests their potential as biomarkers for diagnosing, prognosing, and treating prostatic diseases. These findings offer new insights into the molecular mechanisms linking HPV to prostatic pathologies.

## Simple Summary

Chronic inflammation is a key factor in prostatic diseases, such as prostatitis, benign prostatic hyperplasia (BPH), and prostate cancer (PCa). This study explored the role of human papillomavirus (HPV) infection in these conditions by analyzing the expression of specific microRNAs (miRNAs) linked to various cellular processes involved in cancer. We identified 284 miRNAs associated with PCa by performing in silico analysis; these were found to be involved in cancer development and progression. Notably, a subset of these miRNAs exhibited dysregulated expression in HPV-positive tissues, suggesting their role in promoting a pro-oncogenic environment. These findings identify potential miRNA biomarkers for improving the diagnosis and treatment of prostatic diseases.

## 1. Introduction

Prostate pathologies, including chronic prostatitis, benign prostatic hyperplasia (BPH), and prostate cancer (PCa), are strongly associated with chronic inflammation, which is a key risk factor and hallmark of these diseases [1]. However, the precise molecular mechanisms and cellular pathways driving the progression of these prostate conditions remain largely unclear [2]. Prostatitis, the third most common urological disorder in males, affects 10–14% of men of all ages and ethnic origins and is frequently presented as a comorbidity when diagnosing PCa and BPH [3,4,5,6,7]. BPH’s prevalence rises from 8% at age 40 up to 60% at age 90 [8]. On the other hand, according to the International Agency for Research on Cancer (IARC), in 2020, there were an estimated 1.4 million new cases of PCa worldwide, making it the second most diagnosed cancer in men [9].

In addition to its association with prostate diseases, human papillomavirus (HPV) infection has been implicated in pre-invasive lesions in other tissues, such as high-grade vaginal intraepithelial neoplasia (VaIN2/3) and vulvar squamous cell carcinoma (VSCC). VaIN2/3 is considered a precursor to vaginal cancer, while VSCC is a rare malignancy of the female genital tract. Petri et al. [10] investigated the distribution of HPV genotypes in a large cohort of VaIN2/3 patients in Italy and found HPV DNA in 94.4% of cases, with HPV 16 as the most prevalent genotype, followed by HPV 58, HPV 73, and HPV 31. Interestingly, 73.2% of lesions were associated with a single HPV genotype, predominantly in advanced VaIN3 cases.

Similarly, a study on VSCC in Italian women found that only 7.8% of tumors were associated with mucosal high-risk HPV, with HPV 16 as the most common genotype. This study highlighted the limited role of HPV in VSCC etiology, as only 4.9% of tumors were positive for both viral DNA and RNA, and suggested that beta and gamma HPV types are unlikely to contribute directly to vulvar carcinogenesis [11].

Both studies underscore the importance of HPV vaccination in reducing the incidence of HPV-related lesions in various anatomical sites.

The relationship between the presence of HPV and prostate pathologies is contradictory. Bergh et al. [12] did not find any relationship between the presence of HPV and BPH. Adami et al. [13] and Effert et al. [14] suggest that there is no direct association between HPV types 16 or 18 and PCa; however, they suggest a potential link with HPV type 33.

Contrastingly, other research groups have detected HPV infection in prostate tissue samples, as exemplified by Singh’s study [15]. They analyzed 95 PCa and 55 BPH samples. HPV infection was found in 41% of the prostate tumor biopsies and 20% of the BPH samples. Specifically, 32% of samples were infected with HPV 16 and 6% with HPV 18, while only 5% of BPH samples had HPV 16. A significant proportion of HPV-infected cases were in advanced stages III and IV with high Gleason scores, suggesting that HPV infection may be a cofactor in PCa progression.

Although prostatitis is not considered a premalignant condition, some studies suggest that it could be associated with a slightly increased risk of developing PCa [16,17,18]. However, this relationship remains a subject of debate, and there is no conclusive evidence directly linking it to prostatic carcinogenesis. High-risk HPV infections have been associated with chronic inflammation, which is a critical factor in the development of various types of cancer, including PCa [19]. While the exact role of HPV in prostate diseases is not fully understood, its ability to induce persistent inflammation suggests a potential connection. Nevertheless, it is still unclear how the virus alters specific cellular mechanisms that contribute to inflammatory lesions and hyperproliferation in the prostate gland, or how it may transform normal prostate cells into malignant ones [5,15,20].

Chronic inflammation is regarded as the seventh hallmark of carcinogenesis and as a possible trigger for tumor initiation and progression at the cellular and molecular levels [21]. During pro-tumor inflammation, several inflammatory molecules are either activated or suppressed for a prolonged period of time, leading to undesirable consequences that encourage the growth and enrichment of aggressive tumor phenotypes in the tumor microenvironment [2]. This process stimulates the infiltration of immune cells and the production of chemokines, cytokines, and free radicals, which can damage DNA and activate the androgen receptor (AR) in prostatic cells [22,23,24].

The expression of various micro-RNAs (miRNAs) has been associated with the progression and appearance of different types of cancer, including prostate, breast, lung, liver, gastric, pancreatic, and colorectal cancer, among others [25,26]. Also, several studies have demonstrated the crucial role of miRNAs in the pathogenesis of cancer. Specifically, miR-34a, miR-106a, miR-143, miR-145, and miR-221 have garnered significant attention in cancer biology. These miRNAs have been implicated in regulating critical processes such as cell proliferation, apoptosis, differentiation, inflammatory response, angiogenesis, tumor suppression, adhesion, metastasis, and immune system evasion [25,26,27,28,29,30].

Viral proteins E6 and E7 of HPV play a crucial role in altering genetic regulation in infected cells, specifically affecting miRNAs associated with tumorigenesis. E6 binds to the p53 protein, a key tumor regulator, promoting its degradation and, consequently, disrupting the expression of miRNAs regulated by p53 [31].

Similarly, E7 interacts with the retinoblastoma protein (pRb), a critical regulator of the cell cycle, leading to its inactivation and contributing to uncontrolled cellular proliferation [32,33,34]. In addition to pRb, E7 also binds to other proteins, such as p107 and p130, which are essential for the regulation of cellular proliferation, differentiation, and apoptosis through their interactions with various molecular pathways. Together, the activities of E6 and E7 create a cellular environment conducive to oncogenesis by simultaneously targeting multiple regulatory axes and interfering with the cellular machinery and signaling pathways crucial for cell cycle control and apoptosis [32,33,34].

These disruptions, in turn, alter the expression of several miRNAs, further contributing to tumorigenesis. Both E6 and E7 also interfere with the cellular machinery and cellular signaling pathways important for cell cycle control and apoptosis, which in turn alter the expression of several miRNAs. The interaction of E6 and E7 with c-Myc, p53, and E2F modulates the expression of the miR-15/16 cluster, the miR-17-92 family, miR-21, miR-23b, miR-34a, miR-106b/93/25 cluster, miR-143/145 cluster, and let-7c [35,36]. Furthermore, Chiantore et al. [37] demonstrated that these oncoproteins also influence the expression of miR-18a, miR-19a, miR-34a, and miR-590-5p. They further identified miR-222, a critical miRNA often deregulated in various cancers, in exosomes from HPV-infected cells.

Recent studies have highlighted the importance of nuclear receptors in the modulation of miRNAs in oncological contexts. For instance, it has been observed that the estrogen receptor (ER) inhibits the expression of the *miR-221/222* genes in breast cancer, while in the progression of PCa, *miR-125b*, *miR-21*, and *miR-221/222* could be directly regulated by the androgen receptor (AR) [38].

In Mexico, there is a significant lack of documented studies examining the role of HPV infection and its relationship with the deregulation of miRNAs expression in prostate diseases. Therefore, the present study aimed to analyze the expression profiles of specific miRNAs involved in the pathogenesis of the prostate gland in HPV-positive and HPV-negative biopsy samples with benign (BPH/prostatitis) or malignant (PCa) lesions. These analyses will provide valuable insights into how viral infections and inflammation contribute to the progression of prostate diseases and may help to identify potential miRNAs that could serve as biomarkers for early detection or as therapeutic targets.

## 2. Materials and Methods

### 2.1. Biological Samples

A retrospective analysis was performed on paraffin-embedded prostatic tissue samples sourced from the histopathology archives of the Department of Anatomopathology at ISSSTE Hospital General Dr. Darío Fernández Fierro, covering the period between 2017 and 2019. In our study, we included a total of 94 samples, categorized as follows: 14 healthy tissues (HPV-negative tissue biopsies), 13 samples with BPH, 37 samples with BPH combined with prostatitis, and 30 PCa samples. These cancer samples were stratified according to the Gleason scale: 21 were classified as high-grade malignancies (Gleason score 8–10) and 9 as low-grade malignancies (Gleason score ≤ 6).

Clinical files from the pathology archive were reviewed to discard samples from patients receiving anti-inflammatory drugs, antibiotics, or steroids within six months prior to biopsy. Furthermore, samples from patients who had undergone radiation or chemotherapy, samples with insufficient biological material or poor tissue preservation after collection or processing, or those that failed amplification of internal control during molecular analysis were also excluded.

### 2.2. Ethical Approval and Compliance

The study was approved with the CBE/007/2019 number by the Bioethics Committee of the ENMyH-IPN.

### 2.3. Histopathological Analyses and DNA Extraction

Slight modifications to the protocols described by Pérez et al. [39] and Martínez et al. [40] were made for the processing samples and Hematoxylin–Eosin staining (H&E)). Formalin-fixed paraffin-embedded prostate tissue biopsies were processed using a mechanical tissue processor (Leica Instruments, Nussloch, BW, Germany). Samples were then incubated in xylene and subjected to a series of alcohol washes. The specimens were embedded in paraffin, sectioned at 5 μm, and stained with H&E. Two experienced pathologists examined the slides to confirm the diagnosis of each pathological entity and to determine the severity level of the PCa group using the Gleason scale.

DNA was extracted from the paraffin-embedded tissues using the deparaffinization technique of Johann et al. [6] and the DNeasy Blood & Tissue kit (QIAGEN, Germantown, MD, USA) according to the manufacturer’s recommendations. The extracted DNA samples were quantified at 260 nm using a spectrophotometer (Epoch™, BioTek^®^) (BioTek Instruments, Winooski, VT, USA). Samples with DNA purity of 1.4–1.8 and a DNA concentration of ≥100 ng/μL were used for HPV detection. The DNA quality was determined by amplifying the human β-globin gene (approximately 268 base pairs; bp) using the primers GH20 (5′-GAAGAGCCAAGGACAGGTAC-3′) and PC04 (5′-CAACTTCATCCACGTTCACC-3′).

β-globin was used as an endogenous control to assess the integrity of DNA extraction from the samples. The amplification protocol was performed as described by Zandnia et al. [41] and Williamson et al. [41,42], and consisted of an initial 5 min denaturation step at 95 °C, followed by 30 amplification cycles. Each cycle consisted of 30 s at 95 °C for denaturation, 30 s at 62 °C for annealing, and 30 s at 72 °C for extension. A final extension step of 5 min at 72 °C was applied to complete the amplification. The amplified products were analyzed by electrophoresis in 30% polyacrylamide gels. Only β-globin-positive samples were selected.

### 2.4. HPV Detection by Multiplex PCR

HPV detection was carried out using the GP5+/6+ pair of degenerate oligonucleotides that amplify a 150 bp sequence within the L1 region of the HPV genome. The touchdown protocol described by Evans et al. [43] was followed. To determine the genotype of HPV, the multiplex PCR (mPCR) Human Papilloma Virus Set 2 kit (Maxim Biotech, Rockville, MD, USA) was employed according to the manufacturer’s instructions. This kit was designed specifically for the simultaneous detection of the 8 most prevalent HPV genotypes in the population; 6 and 11 (low risk); 31, 33, 52, and 58 (intermediate risk); and 16 and 18 (high risk).

Each sample was tested in triplicate to ensure the accuracy of the results. PCR products were separated by electrophoresis using 12% polyacrylamide gels and analyzed after staining with ethidium bromide (Bio-Rad, Hercules, CA, USA).

### 2.5. In Situ PCR

To identify the high-risk-HPV (HR-HPV) E6/E7 viral genes within the prostate tissue samples, direct in situ PCR was performed using specific primers, as previously described in the literature [44]. The procedure involved incubation of dried dewaxed sections on DNase/RNase-free electrocharged slides with Proteinase K, followed by washing with ultrapure water. PCR optimal solution containing digoxigenin-11-(2′-deoxy-uridine-5′)-triphosphate, DIG-11-dUTP, (Roche, Indianapolis, IN, USA) was used. In situ PCR was carried out using the system provided by Perkin Elmer, employing a hot start method and two consensus sequence primer pairs within E6 and E7 of HR-HPV (pU-1M and pU-2R primers) [45], and 5 U of Taq DNA polymerase. The cycling conditions included 2 min at 94 °C, followed by 18 cycles of 94 °C for 1 min, 60 °C for 1 min, and 72 °C for 1 min. Clips and AmpliCover discs were then removed, and slides were washed in PBS, followed by 5 min in 100% EtOH, before being air-dried.

### 2.6. HPV Sequence Detection of In Situ PCR Products

To detect the PCR product, an indirect immunolabelling method using a primary anti-digoxigenin antibody (Roche, Indianapolis, IN, USA) conjugated to alkaline phosphatase was employed. Prior to labeling, a blocking step was carried out using 5% BSA (Sigma, St. Louis, MO, USA) in PBS for 30 min. The slides were then drained, and an anti-DIG antibody was added at a dilution of 1:200 in 100 mM Tris HCl pH 7.4 and 150 mM NaCl and incubated for 2 h at room temperature. Detection of alkaline phosphatase was achieved by using the NBT/BCIP kit (Roche, Indianapolis, IN, USA) for 10 min. After detection, the slides were rinsed in distilled water for 5 min and counterstained with Fast Green. Finally, the slides were air-dried before being mounted in Permount histological mounting medium (Fisher Scientific, Waltham, MA, USA).

### 2.7. Digital Analysis and Relative Semi-Quantification of In Situ-Positive Signal

A set of low-amplification (5x) images of up to 10 areas of each tissue sample were captured using a DFC290 HD digital camera (Leica Microsystems, Buffalo Grove, IL, USA). Subsequently, the experimental image files were digitally processed to obtain a uniform signal using PhotoImpact (Ulead PhotoImpact SE Version 3.02, Ulead Systems, Torrance, CA, USA). The relevant regions were digitally assessed using the Image-ProPlus Analysis Software (Version 4.5.0.19, Media Cybernetics, Inc., Rockville, MD, USA). All pixels exhibiting a positive amplification signal for HPV E6/E7 oncogenes, and falling within the specified threshold parameters, were quantified to produce a graphical representation [44].

### 2.8. Interactomic Network and Functional Enrichment Analysis of miRNAs in PCa

To identify the miRNAs involved in PCa, we constructed an interactomic network using the miRNet platform, version 2 (https://www.mirnet.ca/Secure/NetworkBuilder.xhtml, accessed on 15 October 2024). The query was performed using the disease term “prostate cancer” with default parameters, including the consideration of all possible nodes and the generation of a network containing up to 1000 potentially implicated miRNAs.

The term “prostate cancer” retrieves miRNAs with documented involvement in the disease, based on experimental and clinical evidence. miRNet integrates data from validated sources, including miRBase, TarBase, and miRTarHumans, ensuring that the resulting network consists exclusively of miRNAs experimentally associated with PCa. This enhances the reliability and accuracy of the analysis, providing a clear representation of their relevance in this context.

With the aim of obtaining a functional classification of the miRNAs identified in the PCa interactomic network, we performed a second analysis using the miRNet platform. This analysis involved the functional clustering of the miRNAs present in the previously constructed network through enrichment analysis based on curated databases such as the Kyoto Encyclopedia of Genes and Genomes (KEGG), ensuring a robust statistical analysis and biologically meaningful results. For this purpose, we selected the “miRNA Function” option within the Function Explorer tool and applied the Hypergeometric Test.

This statistical test evaluates the significance of functional enrichment by determining whether the number of miRNAs associated with a specific function is greater than expected by chance. As a selection criterion, we considered only the top 10 groups based on the adjusted *p*-value (adj.Pval), which indicates the statistical significance corrected for multiple comparisons. Groups with lower *p*-values represent the most relevant biological functions, as they demonstrate significant enrichment of miRNAs in these categories.

With the obtained results, we plotted the functional groups alongside the number of miRNAs associated with each function, providing a clear visual representation of the most relevant categories in the interactomic network.

### 2.9. RT-qPCR for miRNAs

miRNAs were extracted from 20 µm thick dewaxed tissue sections of biological samples using the standard TRIzol method [46] and conserved in DEPC-treated water to prevent degradation. The concentration of extracted RNA was quantified by a spectrophotometer (Epoch™, BioTek^®^) (BioTek Instruments, Winooski, VT, USA) to ensure a purity of 1.7–1.9 (260/280 ratio) and a concentration of ≥50 ng/µL. The cDNA synthesis (RT) of each miRNA was performed using 50 ng of RNA and the TaqMan^®^ Micro-RNA Reverse Transcription Kit (Applied Biosystems, Foster City, CA, USA) according to the manufacturer’s instructions.

The RT-qPCR assays were carried out using the TaqMan Micro-RNA Assays with the Stratagene system MX 3000p (Thermo Fisher Scientific, Waltham, MA, USA) and included 40 cycles (denaturation at 95 °C, annealing and extension at 60 °C). We used commercial TaqMan probes (Applied Biosystems, Foster City, CA, USA) for the detection of hsa-miR-34a-5p (ID: 000426), hsa-miR-106a-5p (ID: 002169), hsa-miR-143-5p (ID: 002146), hsa-miR-145-5p (ID: 002278), hsa-miR-221-5p (ID: 000524), hsa-miR-222-5p (ID: 002097), hsa-let-7c-5p (ID: 479365_mir), and hsa-miR126-5p (ID; 47888_mir). The internal control RNU48 was used to compare the relative expression of selected miRNAs in experimental tissue samples using the 2-^ΔΔCt^ method [47]. All experiments were performed in triplicate.

### 2.10. Target Gene Prediction for miRNAs in PCa

The prediction of target genes for the differentially expressed miRNAs in prostate cancer was performed using the databases miRDBase (https://mirdb.org/mirdb/index.html, accessed on 18 October 2024), TargetScanHuman (https://www.targetscan.org/vert_80/, accessed on 20 October 2024), and miRWalk (http://mirwalk.umm.uni-heidelberg.de/, accessed on 20 October 2024). Subsequently, the signaling pathways modulated by these target genes, particularly those associated with cancer, were analyzed and identified using gene databases such as GeneCards (https://www.genecards.org/, accessed on 23 October 2024) and Gene Expression Omnibus (GEO) (https://www.ncbi.nlm.nih.gov/geo/, accessed on 23 October 2024).

### 2.11. Statistical Analysis

Graphics were generated using the GraphPad Prism version 8.0.1 (GraphPad Software, San Diego, CA, USA) and IBM SPSS Statistics 25 software (IBM Corporation, Armonk, NY, USA), with a 95% confidence interval. The statistical analyses were performed using multiple *t*-tests per row or two-way ANOVA, applying Tukey’s multiple comparison test. The levels of statistical significance were determined in accordance with the guidelines outlined by the American Psychological Association (APA), with * *p* ≤ 0.033, ** *p* ≤ 0.002, and *** *p* ≤ 0.001. The data presented represent the standard deviation obtained from at least three independent experiments. Finally, differences in intragroup, ∆Ct data were compared using boxplots in the JMP statistical software, version 17 (SAS Institute, Cary, NC, USA).

## 3. Results

### 3.1. Histopathological Analysis of Benign Lesions and PCa

**Figure 1:**
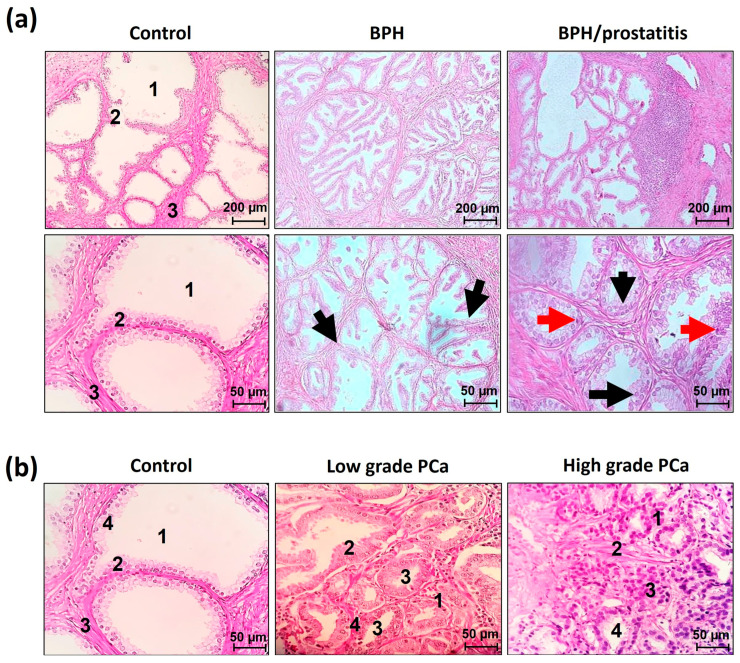
H&E staining for histopathological analysis of benign and PCa biopsy samples. (**a**) Representative images of the tissue architecture of control; benign prostatic hyperplasia (BPH); and BPH plus prostatitis (BPH/prostatitis) samples. Black arrows indicate areas with increased epithelial growth and enlarged nuclei restricted to the basal layer, while red arrows highlight regions with significant inflammatory infiltrates. (**b**) Representative images of prostate tissue architecture in the stratification of low- and high-Gleason-grade PCa. Key tissue structures are shown in both panels,, including the acinar lumen (1), luminal cells (2), stroma (3), and cellular organization (4), are labeled for clarity. Scale bars represent 200 and 50 μm.

Representative images of histopathologic samples are shown in Figure 1. The examination of the control group samples revealed prominent acinar lumens and well-organized epithelial acini, supported by stromal elements, showing a high degree of cellular differentiation and organization.

BPH lesions were primarily located in the transitional and peripheral zones of the prostate gland. An increase in epithelial growth with enlarged nuclei restricted to the basal layer was observed. The glandular sizes varied from medium to large acini and often displayed luminal papillae. A reduction in the acinar lumen was noticeable, caused by the hyperproliferation of luminal cells. This hyperproliferation resulted in a partial loss of cellular homogeneity and a reduction in the stroma, although the cells remained differentiated.

In the BPH/prostatitis group, lesions were identified mainly in the transitional and peripheral zones of the prostate. These lesions were characterized by typical BPH alterations along with multifocal infiltrates of mononuclear cells, including lymphocytes, monocytes, and plasma cells, and an increase in epithelial growth with enlarged nuclei restricted to the basal layer. Some samples exhibited significant inflammatory infiltrates, while others showed both conditions coexisting, with cells displaying prominent nuclei in the basal and suprabasal layers, accompanied by an inflammatory infiltrate.

Inflammation was found at the periglandular level, where a mononuclear leukocyte infiltrate was present adjacent to the prostatic acini, along with a decrease in the intraepithelial component. In some cases, stromal inflammation coexisted with the periglandular inflammation. Important tissue structures, such as the acinar lumen, luminal cells, stroma, and cellular organization, were affected by cellular hyperproliferation and inflammation (Figure 1a).

Regarding PCa, low-Gleason-grade tumors are characterized by the formation of pseudoacinar structures in which the acinar lumen is reduced by hyperproliferation of luminal cells. Such proliferation leads to partial loss of cell homogeneity and stroma reduction, although the cells were still differentiated. Finally, PCa high-Gleason-grade tumors are completely lost their own structures, and hyperproliferation of luminal cells is observed. Basal cells, stroma, and cellular homogeneity are lost. The acinar lumen is almost null, and the cells are poorly differentiated (Figure 1b).

### 3.2. Histological Identification of Koilocytes and In Situ Molecular Detection of HPV Sequences

**Figure 2:**
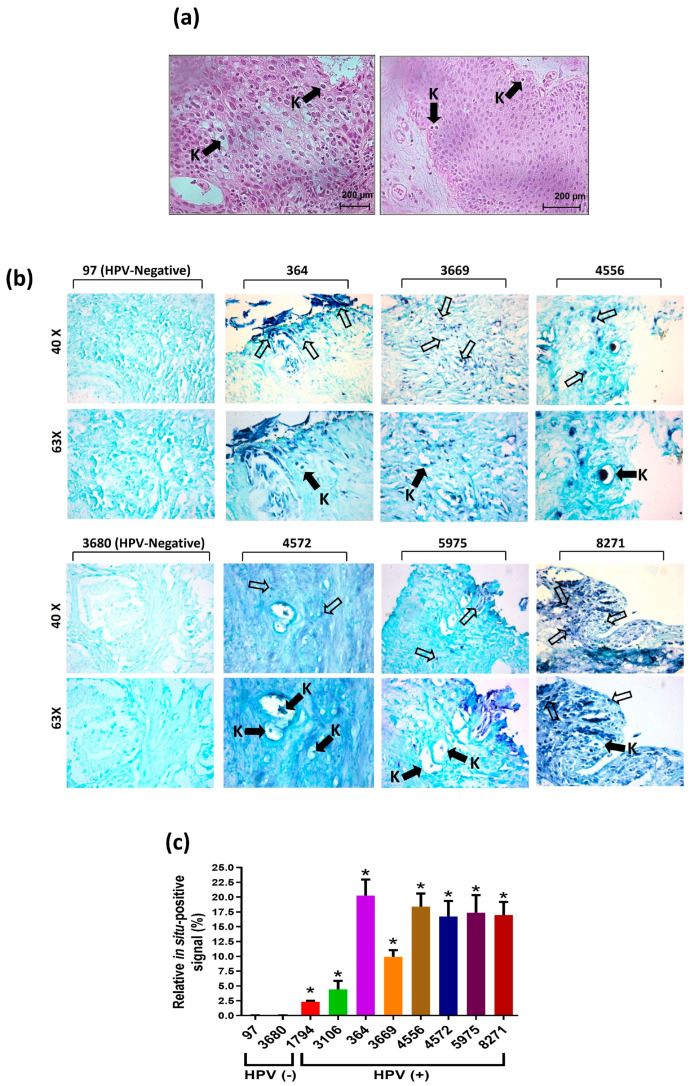
Koilocyte identification and in situ detection of HPV in BPH samples. Panel (**a**) shows the presence of koilocytes (black arrows) identified by H&E staining, and panel (**b**) demonstrates HR-HPV E6/E7 DNA detection via in situ PCR, with signals localized mainly in the nucleus (empty arrows) and in koilocytes (black arrows with “K”). Samples 97 and 3680 served as HPV-negative controls, while samples 364, 3669, 4556, 4572, 5975 and 8271 were HPV-positive. Amplifications: 40× and 63×. Panel (**c**) displays digital quantification of the signal. Statistical analysis: one-way ANOVA with Tukey’s test; results are expressed as mean ± SD, *p* < 0.033 (*).

Histopathological and molecular analyses were conducted to identify the presence of koilocytes or pseudokoilocyte-like structures, recognized as a pathognomonic feature of HPV infection. Representative images of pseudokoilocytes, characterized by enlarged nuclei and perinuclear halos, are displayed in Figure 2a. These structures were predominantly observed in samples positive for HPV genotypes.

To confirm the presence of HPV, in situ PCR for E6/E7 HPV DNA was performed. The results demonstrated a clear and specific detection of viral DNA in HPV-positive samples, particularly in areas exhibiting koilocyte-like morphological changes (Figure 2b). In contrast, HPV-negative samples (97 and 3680) lacked such structures and did not show positive signals.

The in situ semi-quantification of viral presence (Figure 2c) revealed that HPV-positive samples (364, 3669, 4556, 4572, 5975 and 8271) exhibited significantly higher levels of in situ-positive signals compared to HPV-negative samples (97 and 3680). These results demonstrate a clear correlation between the presence of koilocyte-like structures and the detection of HPV DNA.

### 3.3. Multiplex HPV Amplification

To determine the HPV genotype in each sample, a multiplex PCR (mPCR) approach was performed using the Human Papilloma Virus Set 2 kit (Takara Bio, San Jose, CA, USA), which targets the L1 region of the HPV genome. This method allows for the simultaneous detection of 8 prevalent HPV genotypes, including low-risk types (6, 11), intermediate-risk types (31, 33, 52, 58), and high-risk types (16, 18).

The results showed that 67.2% of the samples with benign lesions were positive for any HPV genotype, in contrast to a positive rate of 93.4% in PCa samples. In HPV-positive benign lesions, the predominant genotypes were the low-risk HPV 6 and HPV 11, whereas in malignant lesions, the predominant genotypes were the high-risk HPV 16, 18, 31, 33, 52, and 58.

In PCa samples, 30% of HPV-positive cases were classified as low-grade PCa (Gleason score ≤ 6), with predominant genotypes being HPV 6 and 11, while 70% were classified as high-grade PCa (Gleason score 8–10), with predominant genotypes being HPV 16 and 18. Additionally, high-risk HPVs (HR-HPVs) were mainly present in PCa samples with high Gleason scores, although they were also detected in some low-grade samples, where the most common genotypes were HPV 31 and 52. Interestingly, HR-HPV genotypes were observed in both low- and high-Gleason grade PCa groups (Table 1).

### 3.4. miRNAs Associated with PCa

**Figure 3:**
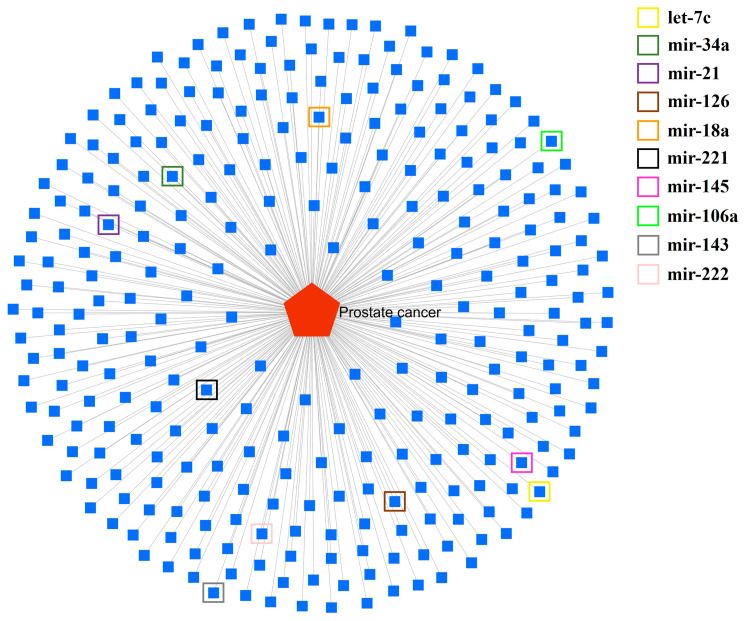
Interactome analysis of miRNAs linked to PCa. The miRNAs highlighted in squares with different colors were selected for expression analysis in our study, based on documented studies supporting their relevance in this pathology.

Through interactomic analysis with miRNet, 284 miRNAs associated with PCa were identified, as illustrated in Figure 3 and listed in Supplementary Table S1. Among them, some key miRNAs, such as let-7c, mir-21, mir-34a, mir-126, mir-18a, mir-145, mir-221, mir-106a, mir-222, and mir-143, were highlighted in the network with colored boxes (Figure 3).

These miRNAs have not only been documented for their involvement in PCa, as shown in Table 2, but also in other types of cancer [25,26,48,49], suggesting their relevance as potential biomarkers or therapeutic targets. Therefore, in our study, they were selected to evaluate their expression in the different experimental groups.

Based on the results obtained and in order to identify the functional clusters associated with the miRNAs in the interactomic network, we performed a functional enrichment analysis. The results obtained show that the 284 miRNAs identified in the interactome network are clustered into key biological processes associated with cancer. Within the top 10, processes such as cell death, cell cycle, apoptosis, tumor suppressors, and hormone-mediated signaling pathways stand out, all with more than 72 miRNAs associated with each cluster. Functional enrichment suggests that these miRNAs play a central role in regulating critical pathways involved in cancer progression and development.

**Figure 4:**
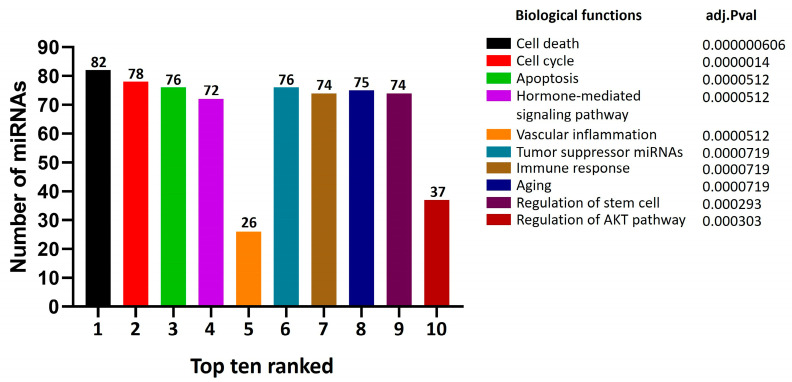
Functional enrichment of miRNAs implicated with PCa. The top ten functional clusters are shown based on interactomic analysis and adjusted *p*-values (adj.Pval). Numbers on the bars indicate the number of miRNAs in each group.

Additionally, processes like aging and stem cell regulation highlight the role of miRNAs in maintaining cellular homeostasis and influencing cancer-related mechanisms. Despite processes such as vascular inflammation (26 miRNAs) and regulation of the AKT pathway (37 miRNAs) having the lowest number of miRNAs in our top 10, their representation remains relevant due to their statistical significance (adjusted *p*-value) and their crucial role in cancer progression, underscoring their importance despite the lower number of associated miRNAs (Figure 4).

### 3.5. Expression Levels of miRNAs in BPH, BPH/Prostatitis and PCa Samples

To identify specific molecular mechanisms that may be altered in benign lesions of the prostate gland, we conducted a study using RT-qPCR to evaluate the expression profiles of six miRNAs (miR-34a, miR-106a, miR-143, miR-145, miR-221, and miR-222). These miRNAs were selected based on an interactomic analysis and on their established roles in the pathogenesis of prostatic inflammation and cell proliferation [28,49]. The miRNAs were evaluated in HPV-negative or -positive, BPH or BPH/prostatitis samples.

**Figure 5:**
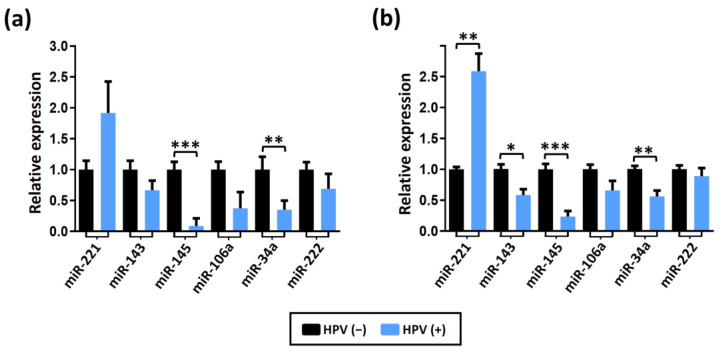
miRNAs expression levels in HPV-negative (−) or HPV-positive (+) benign prostatic samples. Expression of indicated miRNAs in BPH (**a**) or in BPH/prostatitis (**b**) is shown. The expression data were normalized using RNU48 as an internal control. Statistical analysis was performed using multiple *t*-tests per row. The error bars represent the standard deviation. Three levels of significance were used for *p*-values: * *p* < 0.033, ** *p* < 0.002, and *** *p* < 0.001.

The results showed a decrease in the expression of miR-145 and miR-34a in HPV-positive samples from both BPH (Figure 5a) and BPH/prostatitis (Figure 5b), compared to HPV-negative samples. Interestingly, we observed a significant increase in the expression of miR-221 and a decrease in miR-143 specifically in HPV-positive BPH/prostatitis samples, compared to HPV-negative samples. In contrast, these two miRNAs (miR-221 and miR-143) did not show significant changes in expression in BPH samples. Finally, miR-222 did not exhibit significant alterations in its expression in either BPH or BPH/prostatitis samples.

**Figure 6:**
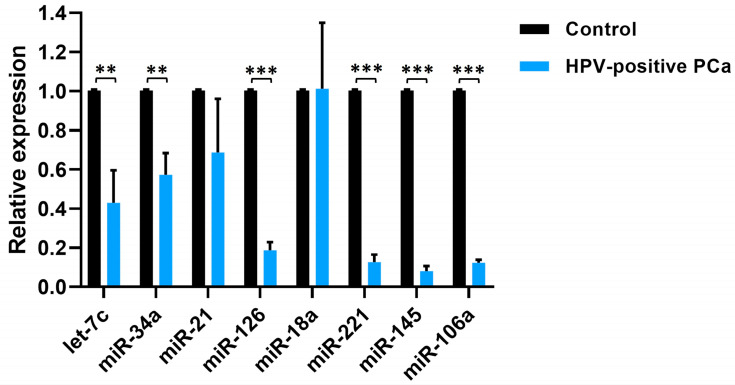
miRNAs expression levels in HPV-positive PCa samples compared to their expression in control prostate tissue. Statistical analysis was performed using multiple T-tests per row. The error bars represent the standard deviation. Three levels of significance were employed for *p*-values: ** *p* < 0.002, and *** *p* < 0.001.

For the analysis of the miRNAs expression profile of PCa, in addition to miR-34a, miR-221, miR-145, and miR-106a, we included the miRNAs let-7c, miR-21 and miR-126, and miR-18a involved in the PCa tumorigenesis [28]. In HPV-positive PCa samples, we observed a significant decrease in the expression levels of let-7c, miR-34a, miR-126, miR-221, miR-145, and miR-106a compared to the control group. In contrast, no significant differences in the expression levels of miR-21 and miR-18a were observed (Figure 6).

We then performed two comparative analyses of the relative expression of miRNAs in HPV-positive PCa samples, which were classified by Gleason histopathological grade as high (8–10) or low (≤6). The first analysis compared these samples with HPV-negative PCa samples, while the second analysis evaluated the differences between low- and high-grade HPV-positive samples.

**Figure 7:**
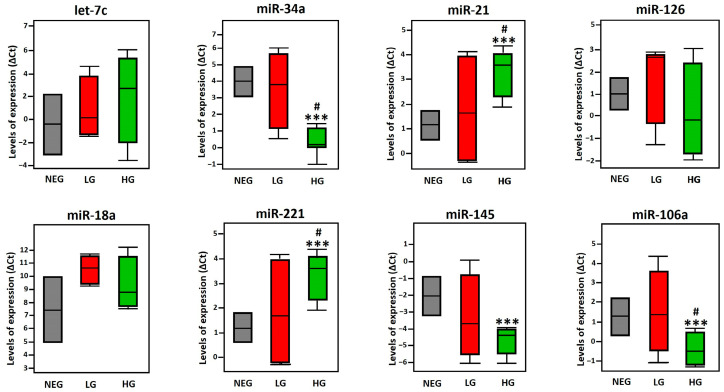
Evaluation of miRNA expression levels in HPV-positive and HPV-negative PCa samples. The expression levels of miRNAs were determined in HPV-positive PCa samples of high grade (HG) and low grade (LG), as well as in HPV-negative PCa samples (NEG). Statistical analyses were performed using two-way ANOVA with Tukey’s multiple comparison test. *p*-values indicated statistically significant differences between the groups, where *** *p* < 0.001 wasused for comparisons between LG, HG, and NEG. Meanwhile, the symbol # was used to indicate *p* < 0.033 for direct comparisons between LG and HG groups.

The results showed a significant decrease in the expression of miR-34a, miR-145, and miR-106a in high-grade (HG) PCa samples compared to HPV-negative PCa samples (NEG). In contrast, miR-21 and miR-221 exhibited increased expression in HG samples relative to NEG. Interestingly, miR-21 and miR-221 were the only miRNAs that showed an increase in HG samples compared to low-grade (LG) PCa samples, while miR-106a showed a significant decrease between these two groups. Finally, Let-7c, miR-126, and miR-18a did not show significant changes in their expression levels relative to NEG or between LG and HG samples (Figure 7).

The statistical difference observed in the expression of miRNAs in HG PCa samples, both compared to NEG and LG PCa samples, suggests that the expression of viral oncoproteins may be involved in the dysregulation of these miRNAs and possibly in the carcinogenesis of the prostate gland.

### 3.6. Identification of Gene Targets and Key Cellular Processes Regulated by miRNAs Associated with Cancer

To evaluate the gene targets and key cellular processes modulated by differentially expressed miRNAs in high-grade PCa samples, according to the Gleason scale, we predicted the gene targets and cancer-related cellular processes.

We found that miR-34a can regulate the genes UHRF2, MDM4, and MET; miR-145 targets the genes SOX11, ERG, MYO6, GMFB, and BCR; miR-221 targets FOXS1, KIT, DMTF1, SORCS1, and CCND1; miR-21 targets STAT3, SKP2, CREBRF, and MALT1; and miR-106a targets TP53, RB1, E2F1, and BCL2L11 (Bim), which are closely associated with the modulation of processes such as proliferation, regulation of DNA methylation, genomic stability, DNA damage response, TP53 regulation, cell cycle arrest and evasion, differentiation, migration, survival, resistance to apoptosis, cancer progression, malignancy, angiogenesis, metastasis, inflammatory response, and therapy resistance (Table 3). These processes represent essential cellular functions that may be altered and contribute to tumor cell aggressiveness in advanced PCa.

## 4. Discussion

Chronic inflammation activates pro-inflammatory and oncogenic genes, leading to genomic instability and fostering a microenvironment that is conducive to carcinogenesis. The frequent occurrence of prostatitis in patients diagnosed with PCa underscores the crucial role that chronic inflammation plays in cancer cell growth [2]. In PCa, human papillomavirus (HPV) could exacerbate tumorigenesis by modulating specific miRNAs through its E6 and E7 oncoproteins, influencing key oncogenic pathways [37,38,75]. The objective of this study was to evaluate the expression profiles of a set of miRNAs involved in inflammation, proliferation and oncogenesis in prostate diseases in HPV-positive biopsy samples with BPH/prostatitis or PCa.

The molecular analysis of HPV revealed a significant relationship with benign (BPH, prostatitis/BPH) and malignant (PCa) diseases. While in benign lesions, the percentage of HPV positivity was lower and predominated the low- and intermediate- risk genotypes, the HPV positivity percentage in PCa samples was noticeably higher, particularly in infections of intermediate- and high-risk genotypes, alone or in coinfection with other genotypes, including low-risk genotypes. We found that samples with a Gleason score of ≥8 were highly correlated with the presence of the high-risk virus. Furthermore, the most frequent high-and intermediate-risk genotypes associated with PCa were 16, 31, 33, and 52. Interestingly, the low-risk genotype 6 was frequently found in coinfection with high- or intermediate-risk genotypes.

According to Singh et al. [15], HPV-16 is the most prevalent genotype found in PCa samples. However, they also identified genotypes 31 and 52 in these samples. High-risk HPV genotypes can lead to malignant tumors even in low viral concentrations. Nonetheless, HPV-16 can reach viral loads much higher than other high-risk genotypes, and thus it shows a correlation with the severity of the disease in cervical cancer [76].

Similarly, in our study, PCa samples with high severity (a Gleason score over 8) representing 70%, were infected with HPV-16 and HPV-18. In contrast, it is well known that although low-risk HPV genotypes have no oncogenic effect, they promote persistent inflammation [77]. This asseveration concurs with our findings in benign lesions, BPH and BPH/prostatitis, in which low-risk genotypes 6 and 11 were found in 67% of samples. Chronic inflammation is associated with higher rates of cellular mutations and genetic alterations, which could include tumorigenesis [2].

Additionally, HPV-6 could contribute to this process by promoting a persistent inflammatory microenvironment that facilitates the entry and persistence of high-risk genotypes [78]. Additionally, HPV-6 may modulate the local immune response, reducing the immune pressure on oncogenic genotypes and enhancing their persistence [79]. Synergistic interactions between low- and high-risk genotypes may amplify the disruption of critical molecular pathways, such as miRNA regulation, apoptosis, and angiogenesis, creating a favorable environment for lesion progression [80,81].

Recent studies have further highlighted that coinfections involving both high- and low-risk HPV genotypes can significantly influence the progression of premalignant lesions, such as CIN2, through cooperative oncogenic mechanisms. Collectively, these findings suggest that HPV-6 may act as a facilitator in coinfections, driving processes like chronic inflammation and immune modulation that support the persistence and oncogenic activity of high-risk genotypes [82]. Epidemiological data indicate that chronic infections and inflammation are linked to over 25% of all cancers [83], a finding consistent with the observations made by García-Lozano et al. [84] in relation to cervical cancer.

In this study, 284 miRNAs associated with PCA were identified through an interactome analysis. Complementarily, the functional enrichment analysis revealed that the 284 miRNAs are grouped into key biological processes relevant to cancer progression. These include cell death, cell cycle regulation, apoptosis, hormone-mediated signaling pathways, vascular inflammation, tumor suppressor pathways, immune response, aging, stem cell regulation, and regulation of the AKT pathway, underscoring their crucial role in cancer development and progression. These findings are consistent with different reports from multiple authors who have studied PCa [4,25,26,28,29,30,38,49,54,85].

Among these, key miRNAs, such as let-7c, miR-21, miR-34a, miR-126, miR-18a, miR-145, miR-221, miR-106a, miR-222, miR-143, and miR-106a were selected due to their known roles in modulating various cellular processes, including inflammation, proliferation, cell cycle regulation, metastasis, and the development and progression of cancer, among other fundamental processes, as documented by different authors and mentioned in Table 2. Notably, they have also been associated with HPV infection in other studies, such as in cervical cancer [37,84,86,87,88].

Interestingly, our evaluation revealed a significant downregulation (*p* < 0.05) of let-7c, miR-34a, miR-145, miR-106a, and miR-126 in high-grade PCa samples infected with high-risk HPV genotypes, compared to control prostate tissues. Notably, miR-221, miR-145, miR-126, and miR-106a exhibited the most significant differences (*p* < 0.001), indicating a substantial alteration in the expression of these miRNAs in severe tumor samples. This suggests that HPV infection may induce alterations in these miRNAs, potentially contributing to the oncogenic process.

Interestingly, the histopathological results of the evaluated samples correlate with the miRNA expression profiles observed across different groups of prostatic pathology. In BPH and BPH/prostatitis samples, where epithelial hyperproliferation and a reduction in acinar lumen were noted, a decrease in the expression of tumor-suppressing miRNAs, such as miR-34a and miR-145, was identified. This suggests that the characteristic cellular hyperproliferation in these lesions may be facilitated by the downregulation of these miRNAs, thereby fostering an environment conducive to chronic inflammation.

Additionally, the inflammatory infiltrate observed in the BPH/prostatitis samples correlates with the overexpression of miR-221, a pro-inflammatory miRNA, suggesting that the inflammation in these lesions may be modulated by miRNA-mediated mechanisms. This correlation between structural tissue alterations and miRNA expression profiles further supports the idea that the disruption of the prostatic microenvironment through inflammatory and hyperproliferative processes is crucial for the potential progression towards PCa in HPV-positive patients. Understanding this relationship is vital for uncovering the mechanisms underlying prostate disease progression, as detailed by Lo et al. [49].

The miRNAs let-7c, miR-34a, miR-221, miR-145, and miR-106a are associated with both PCa progression and HPV infection, which contributes to inflammation and cancer development. For example, let-7c is thought to act as a tumor suppressor in various cancers, including PCa, by targeting genes like NRAS and c-Myc. Decreased let-7c expression in PCa cells may lead to increased oncogene activity, promoting proliferation and invasion [26,51,89,90,91,92,93]. Similarly, miR-34a is a tumor suppressor, and its reduced levels may be linked to the increased expression of genes associated with growth and immune evasion. Moreover, miR-34a is considered a promising therapeutic target; it inhibits PCa development, survival, and invasiveness by repressing multiple targets, including the oncogene c-Myc and the androgen receptor [54].

Additionally, the loss of miR-34a has been associated with overexpression of AR and Notch-1, often seen in high-grade lesions [85]. Since miR-34a expression is p53-dependent, and p53 levels are typically lower in cancer cells, this results in reduced miR-34a levels. HPV oncoproteins E6 and E7 can further inhibit miR-34a expression, contributing to overexpression of its target molecules [86,94].

miR-21 plays an important role in PCa progression by regulating apoptosis, promoting cell proliferation, aiding in invasion and metastasis, and potentially contributing to therapeutic resistance. It also modulates the tumor microenvironment and immune response by influencing cytokines and inflammatory factors [95]. In this study, decreased miR-34a levels and increased miR-21 levels were observed in samples with high-Gleason-grade PCa, which was consistent with the findings of Khatami et al. [87], further supporting the role of miR-34a in the development of PCa. Additionally, Stafford et al. [96] found that elevated miR-21 expression is linked to advanced PCa stages, indicating its potential as a prognostic biomarker.

miR-126, which is downregulated in PCa, is involved in processes such as apoptosis, migration, and metastasis by targeting molecules like SIRT1, CDK6, and integrins. Its underexpression is also associated with increased inflammation [59,97,98,99], suggesting that altered miRNA expression, particularly miR-34a and miR-126, may connect inflammatory processes to cancer progression [48]. miR-221 inhibits SOCS3 and IRF2, thereby suppressing the JAK/STAT pathway, which is crucial for the antiviral response to HPV [100,101,102]. The downregulation of miR-221 in PCa could activate the TGFβ pathway and is associated with AR overexpression, promoting cancer progression [103]. However, normal miR-221 levels may inhibit IRF2 and SOCS3, potentially controlling proliferation in androgen-independent PCa [104].

miR-145 functions as a tumor suppressor by regulating the cell cycle and apoptosis, and its underexpression is correlated with PCa progression and the presence of the TMPRSS2-ERG fusion protein [105,106]. It also plays a role in anti-inflammatory processes by inhibiting CD40, IL-6, and CXCL8 [107]. HR-HPV oncoproteins E6 and E7 promote its downregulation to support viral replication [108]. The subexpression of miR-145 has been observed in HPV-associated oropharyngeal carcinoma and inflammatory lesions in PCa, underscoring its importance in tumor formation [109].

Lastly, Shen et al. [4] found that low miR-106a levels are associated with elevated IL-8 in PCa, a cytokine that promotes tumorigenesis and chronic inflammation. HPV oncoproteins increase IL-8 expression, which may explain the downregulation of miR-106a in HPV-positive PCa samples [5]. In summary, the deregulation of miR-34a, miR-221, miR-145, and miR-106a in both inflammatory lesions and HPV-positive PCa suggests their involvement in creating a pro-oncogenic microenvironment.

Complementarily, by predicting target genes of the deregulated miRNAs let-7c, miR-21, miR-34a, miR-126, miR-18a, miR-145, miR-221, miR-106a, miR-222, miR-143, and miR-106a in high-malignancy PCa, we identified potential modulation of genes such as UHRF2, MDM4, MET, SOX11, ERG, MYO6, GMFB, BCR, FOXS1, KIT, DMTF1, SORCS1, CCND1, STAT3, SKP2, CREBRF, MALT1, TP53INP1, RB1, E2F1, and BCL2L11 (Bim). These genes are intricately linked to the regulation of processes like cell proliferation, DNA methylation, genomic stability, DNA damage response, TP53 regulation, cell cycle arrest and evasion, differentiation, migration, survival, apoptosis resistance, cancer progression, malignancy, angiogenesis, metastasis, inflammatory response, and therapy resistance. These findings are consistent with the biological processes identified through the functional enrichment analysis of miRNAs derived from the interactome analysis. Collectively, these cellular functions may be altered, contributing to tumor aggressiveness in advanced PCa.

Ongoing studies will elucidate whether these miRNAs are potential candidates as biomarkers for prognosis, diagnosis, or therapeutic targets in HPV-associated PCa. In parallel, we are conducting assays to confirm the predicted target genes and proteins identified in this study, aiming to strengthen their link to the molecular mechanisms underlying the development and progression of PCa. These efforts are essential not only to establish the clinical relevance of these findings but also to pave the way for novel therapeutic strategies in the management of this disease.

**Figure 8:**
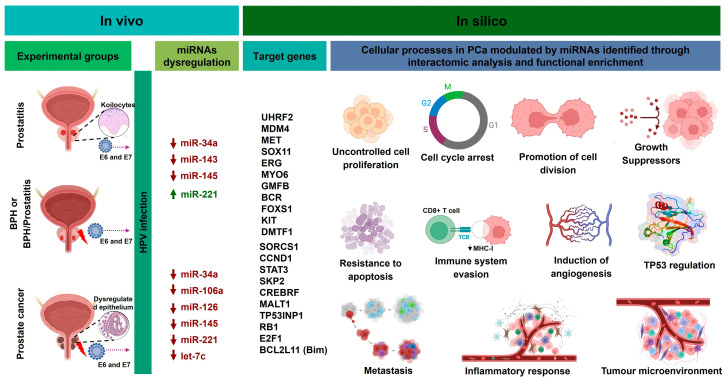
Proposed mechanism of HPV-induced miRNAs dysregulation in benign lesions and PCa, promoting a pro-oncogenic environment and influencing key genes and cellular processes in cancer progression. Upward arrows indicate overexpression and downward arrows indicate downregulation based on our studies; target genes and cellular processes were predicted in silico.

In summary, in Figure 8, we propose a potential mechanism where HPV infection could induce the dysregulation of certain miRNAs in benign and chronic inflammatory lesions, as well as in PCa, promoting a pro-oncogenic microenvironment and altering critical molecular pathways. These dysregulated miRNAs may play a role in the malignant transformation of the prostate gland by modulating genes and cellular processes related to cancer progression.

## 5. Conclusions

This study reveals that HPV infections may contribute to the development and progression of prostatic diseases in Mexico through the deregulation of several miRNAs. In conditions such as BPH and prostatitis, miR-34a, miR-143, and miR-145 were downregulated, while miR-221 was overexpressed. In PCa, miRNAs such as let-7c, miR-34a, miR-126, miR-221, miR-145, and miR-106a exhibited differential expressions, which varied with tumor severity. Additionally, in silico analyses suggest that these miRNAs and their target genes are involved in key biological processes, including cell cycle regulation, apoptosis, angiogenesis, immune response, metastasis, and tumor suppressor pathways, highlighting their role in cancer progression. Our study provides valuable insights into the molecular mechanisms linking HPV infection to prostatic diseases, opening up new perspectives for understanding the role of miRNAs in these pathologies. Furthermore, it suggests that these miRNAs could serve as potential biomarkers for early diagnosis, prognosis, and the development of targeted therapies.

## Figures and Tables

**Table 1:** HPV genotype frequency in benign and PCa lesions, according to the type and degree of injury to the prostate gland.

HPV Genotypes in Benign Lesions Group			
**Type of Injury**	HPV Positivity Frequency (%)	Predominant HPV Genotypes	Coinfection ^[1]^
BPH	LR: 62.5	6, 11	ND
	IR: 25.0	52, 58	6, 11
	HR: 12.5	16, 18	6, 11, 33
BPH/Prostatitis	LR:74	6, 11	ND
	IR: 21.7	52, 58	6, 11
	HR: 4.3	16, 18	6, 11, 33
**HPV Genotypes in PCa Group**			
**HPV Genotypes Depending on PCa Gleason Stratification**			
**Type of Injury**	**HPV Positivity Frequency (%)**	**Predominant HPV Genotypes**	**Coinfection ^[1]^**
Low-grade Gleason (score ≤ 6)	LR-HPV: 55.5	6, 11	58, 52 ^[1]^
	IR-HPV: 11.1	52	16
	HR-HPV: 33.3	16	ND
High-grade Gleason (8–10)	LR-HPV: 10.6	6, 11, 31	31, 52
	IR-HPV: 47.3	33, 31, 52	18
	HR-HPV: 42.10	16, 18	6

^[1]^ In benign lesions, BPH and BPH/prostatitis, coinfections between low- (6 and 11) and intermediate-risk (52, 58, and 33) genotypes were found. In contrast, in PCa lesions, a higher frequency of coinfections between intermediate (31, 33, 58 and 52) and high-risk (16 and 18) genotypes was observed. ND: not detected. LR- and HR-HPV are both low and high HPV genotypes, respectively.

**Table 2:** Biological functions and molecular pathways of selected miRNAs in PCa for experimental validation.

miRNAs	Biological Function	Molecular Pathways Involved	References
let-7c	Promotes cell cycle progression, proliferation, survival, cancer growth, metastasis, and stemness, while reducing therapy resistance.	Regulates Ras, NFκB, and Myc signaling pathways.	Nadiminty et al. [50], Mulholland et al. [51], Reis et al. [52].
miR-34a	Drives proliferation, invasiveness, stemness, and survival. Suppresses apoptosis and accelerates tumor progression.	Modulates p53, c-Myc, and androgen receptor.	Abdelaal et al. [53], Li et al. [54], Liu et al. [55].
mir-21	Enhances cell survival, proliferation, invasiveness, and chemoresistance.	Controls PTEN, PDCD4, and TIMP3 pathways.	Singh et al. [56], Gunawan et al. [57].
mir-126	Modulates angiogenesis, limits tumor growth, and prevents metastasis.	Modulates VEGF, PI3K/Akt, and EGFL7 pathways.	Hua et al. [58], Jalil et al. [59], Sun et al. [60].
mir-18a	Supports proliferation, survival, angiogenesis, invasion, and metastasis, while inhibiting apoptosis in cancer.	Influences Myc, HIF-1α, and p53 pathways.	Hsu et al. [61], Santos et al. [62], Shen et al. [63].
mir-221	Regulates proliferation, survival, migration, invasion, angiogenesis, chemoresistance, and tumor progression.	Modulates p27, p57, and Bim pathways.	Kiener et al. [64], Baruah et al. [65], Goto et al. [66].
mir-145	Acts as a tumor suppressor in cancer. It inhibits proliferation, migration, invasion, angiogenesis, and stemness, while promoting apoptosis.	Targets c-Myc, KRAS, and SOX2 pathways.	Wu et al. [67], Manvati et al. [68], Zeinali et al. [69].
mir-106a	Drives proliferation, survival, migration, invasion, and chemoresistance.	Modulates p21, PTEN, and Bim pathways.	Shen et al. [4], Lu et al. [70], Coman et al. [71].
mir-222	Enhances proliferation, survival, migration, invasion, angiogenesis, and chemoresistance.	Regulates p27, p57, and Bim pathways.	Garofalo et al. [72], Song et al. [73], Wang et al. [74].

**Table 3:** Gene targets and key cellular processes modulated by miRNAs involved in cancer.

miRNAs	Target Genes	Cellular Processe
miR-34a	UHRF2	Proliferation, regulation of DNA methylation, genomic stability, and response to DNA damage.
	MDM4	Regulates TP53, inhibits cell cycle arrest and apoptosis mediated by p53/TP53 and TP73/p73, and inhibits the degradation of MDM2.
	MET	Growth, survival, cell migration, invasion, cancer progression, and malignancy.
miR-145	SOX 11	Regulates cell proliferation, differentiation, and migration.
	ERG	Proliferation, differentiation, and apoptosis.
	MYO6	Cell migration and invasion.
	GMFB	Proliferation, migration, inflammation, and apoptosis.
	BCR	Cell signaling, proliferation, differentiation, survival, migration, invasion, and apoptosis.
miR-221	FOXS1	Proliferation, migration, invasion, and resistance to apoptosis.
	KIT	Cell survival and proliferation.
	DMTF1	Senescence, apoptosis, cell cycle regulation, and DNA damage response.
	SORCS1	Cell proliferation, migration and invasion, and resistance to apoptosis.
	CCND1	Cell cycle during the G1/S transition, proliferation, evasion of apoptosis, therapy resistance, and invasion.
miR-21	STAT3	Proliferation, invasion, metastasis, angiogenesis, evasion of apoptosis, and therapy resistance.
	SKP2	Cell cycle progression through negative regulation of p27, resistance to apoptosis, invasion, and migration.
	CREBRF	Proliferation, survival, and resistance to apoptosis.
	MALT1	Proliferation, survival, resistance to apoptosis, and inflammatory response.
miR-106a	TP53	Apoptosis, cell cycle regulation, DNA damage response, oxidative stress control, inhibition of invasion and metastasis.
	RB1	Cell cycle, proliferation, senescence, apoptosis, maintenance of genomic stability, invasion, and metastasis.
	E2F1	Cellular proliferation, cell cycle regulation, apoptosis, differentiation, DNA damage response, invasion, and metastasis.
	BCL2L11 (Bim)	Apoptosis, cell cycle, cellular stress response and sensitivity to therapies.

## Data Availability

The datasets used and analyzed during the current study are available from the corresponding author upon reasonable request.

## Supplementary Materials

The following supporting information can be downloaded at https://www.mdpi.com/article/10.3390/cancers17010026/s1, Table S1: Interactome analysis of miRNAs implicated in prostate cancer.

## References

[1] Pelucchi C., Talamini R., Negri E., Franceschi S., La Vecchia C. (2006). Genital and Urinary Tract Diseases and Prostate Cancer Risk. Eur. J. Cancer Prev. Off. J. Eur. Cancer Prev. Organ. ECP.

[2] Oseni S.O., Naar C., Pavlović M., Asghar W., Hartmann J.X., Fields G.B., Esiobu N., Kumi-Diaka J. (2023). The Molecular Basis and Clinical Consequences of Chronic Inflammation in Prostatic Diseases: Prostatitis, Benign Prostatic Hyperplasia, and Prostate Cancer. Cancers.

[3] Dai X., Fang X., Ma Y., Xianyu J. (2016). Benign Prostatic Hyperplasia and the Risk of Prostate Cancer and Bladder Cancer: A Meta-Analysis of Observational Studies. Medicine.

[4] Shen P., Sun G., Zhao P., Dai J., Zhang X., Zhao J., Zhu S., Chen J., Tao R., Yang J. (2020). MicroRNA-106a Suppresses Prostate Cancer Proliferation, Migration and Invasion by Targeting Tumor-Derived IL-8. Transl. Cancer Res..

[5] Shiau M.-Y., Fan L.-C., Yang S.-C., Tsao C.-H., Lee H., Cheng Y.-W., Lai L.-C., Chang Y.-H. (2013). Human Papillomavirus Up-Regulates MMP-2 and MMP-9 Expression and Activity by Inducing Interleukin-8 in Lung Adenocarcinomas. PLoS ONE.

[6] Johann D.J., Shin I.J., Roberge A., Laun S., Peterson E.A., Liu M., Steliga M.A., Muesse J., Emmert-Buck M.R., Tangrea M.A. (2022). Effect of Antigen Retrieval on Genomic DNA from Immunodissected Samples. J. Histochem. Cytochem..

[7] Zhang L., Wang Y., Qin Z., Gao X., Xing Q., Li R., Wang W., Song N., Zhang W. (2020). Correlation between Prostatitis, Benign Prostatic Hyperplasia and Prostate Cancer: A Systematic Review and Meta-Analysis. J. Cancer.

[8] Lim K.B. (2017). Epidemiology of Clinical Benign Prostatic Hyperplasia. Asian J. Urol..

[9] International Agency for Research on Cancer (2020). Cancer Today. https://gco.iarc.who.int/today/.

[10] Preti M., Boldorini R., Gallio N., Cavagnetto C., Borella F., Pisapia E., Ribaldone R., Bovio E., Bertero L., Airoldi C. (2024). Human Papillomavirus Genotyping in High-Grade Vaginal Intraepithelial Neoplasia: A Multicentric Italian Study. J. Med. Virol..

[11] Preti M., Rotondo J.C., Holzinger D., Micheletti L., Gallio N., McKay-Chopin S., Carreira C., Privitera S.S., Watanabe R., Ridder R. (2020). Role of Human Papillomavirus Infection in the Etiology of Vulvar Cancer in Italian Women. Infect. Agent. Cancer.

[12] Bergh J., Marklund I., Gustavsson C., Wiklund F., Grönberg H., Allard A., Alexeyev O., Elgh F. (2007). No Link between Viral Findings in the Prostate and Subsequent Cancer Development. Br. J. Cancer.

[13] Adami H.-O., Kuper H., Andersson S.-O., Bergström R., Dillner J. (2003). Prostate Cancer Risk and Serologic Evidence of Human Papilloma Virus Infection: A Population-Based Case-Control Study. Cancer Epidemiol. Biomark. Prev..

[14] Effert P.J., Frye R.A., Neubauer A., Liu E.T., Walther P.J. (1992). Human Papillomavirus Types 16 and 18 Are Not Involved in Human Prostate Carcinogenesis: Analysis of Archival Human Prostate Cancer Specimens by Differential Polymerase Chain Reaction. J. Urol..

[15] Singh N., Hussain S., Kakkar N., Singh S.K., Sobti R.C., Bharadwaj M. (2015). Implication of High Risk Human Papillomavirus HR-HPV Infection in Prostate Cancer in Indian Population- A Pioneering Case-Control Analysis. Sci. Rep..

[16] Nickel J.C., True L.D., Krieger J.N., Berger R.E., Boag A.H., Young I.D., participating members of the North American Chronic Prostatitis Collaborative Research Network and the International Prostatitis Collaborative Network (see) (2001). Consensus Development of a Histopathological Classification System for Chronic Prostatic Inflammation. BJU Int..

[17] Lehrer S., Diamond E.J., Mamkine B., Droller M.J., Stone N.N., Stock R.G. (2005). C-Reactive Protein Is Significantly Associated with Prostate-Specific Antigen and Metastatic Disease in Prostate Cancer. BJU Int..

[18] Yli-Hemminki T.H., Laurila M., Auvinen A., Määttänen L., Huhtala H., Tammela T.L.J., Kujala P.M. (2013). Histological Inflammation and Risk of Subsequent Prostate Cancer among Men with Initially Elevated Serum Prostate-Specific Antigen (PSA) Concentration in the Finnish Prostate Cancer Screening Trial. BJU Int..

[19] Ahmed M.Y., Salman N.A., Sandhu S., Cakir M.O., Seddon A.M., Kuehne C., Ashrafi G.H. (2023). Detection of High-Risk Human Papillomavirus in Prostate Cancer from a UK Based Population. Sci. Rep..

[20] Russo G.I., Calogero A.E., Condorelli R.A., Scalia G., Morgia G., La Vignera S. (2020). Human Papillomavirus and Risk of Prostate Cancer: A Systematic Review and Meta-Analysis. Aging Male.

[21] Coussens L.M., Werb Z. (2002). Inflammation and Cancer. Nature.

[22] Naiyila X., Li J., Huang Y., Chen B., Zhu M., Li J., Chen Z., Yang L., Ai J., Wei Q. (2023). A Novel Insight into the Immune-Related Interaction of Inflammatory Cytokines in Benign Prostatic Hyperplasia. J. Clin. Med..

[23] Ullah A., Chen Y., Singla R.K., Cao D., Shen B. (2024). Pro-Inflammatory Cytokines and CXC Chemokines as Game-Changer in Age-Associated Prostate Cancer and Ovarian Cancer: Insights from Preclinical and Clinical Studies’ Outcomes. Pharmacol. Res..

[24] Hatano K., Fujita K., Nonomura N. (2020). Application of Anti-Inflammatory Agents in Prostate Cancer. J. Clin. Med..

[25] Coradduzza D., Solinas T., Balzano F., Culeddu N., Rossi N., Cruciani S., Azara E., Maioli M., Zinellu A., De Miglio M.R. (2022). miRNAs as Molecular Biomarkers for Prostate Cancer. J. Mol. Diagn..

[26] Smolarz B., Durczyński A., Romanowicz H., Szyłło K., Hogendorf P. (2022). miRNAs in Cancer (Review of Literature). Int. J. Mol. Sci..

[27] Pekarek L., Torres-Carranza D., Fraile-Martinez O., García-Montero C., Pekarek T., Saez M.A., Rueda-Correa F., Pimentel-Martinez C., Guijarro L.G., Diaz-Pedrero R. (2023). An Overview of the Role of MicroRNAs on Carcinogenesis: A Focus on Cell Cycle, Angiogenesis and Metastasis. Int. J. Mol. Sci..

[28] Vanacore D., Boccellino M., Rossetti S., Cavaliere C., D’Aniello C., Di Franco R., Romano F.J., Montanari M., La Mantia E., Piscitelli R. (2017). Micrornas in Prostate Cancer: An Overview. Oncotarget.

[29] Schitcu V.H., Raduly L., Nutu A., Zanoaga O., Ciocan C., Munteanu V.C., Cojocneanu R., Petrut B., Coman I., Braicu C. (2022). MicroRNA Dysregulation in Prostate Cancer. Pharmacogenom. Pers. Med..

[30] Abudoubari S., Bu K., Mei Y., Maimaitiyiming A., An H., Tao N. (2023). Preliminary Study on miRNA in Prostate Cancer. World J. Surg. Oncol..

[31] Tomaić V. (2016). Functional Roles of E6 and E7 Oncoproteins in HPV-Induced Malignancies at Diverse Anatomical Sites. Cancers.

[32] Mir B.A., Ahmad A., Farooq N., Priya M.V., Siddiqui A.H., Asif M., Manzoor R., Ishqi H.M., Alomar S.Y., Rahaman P.F. (2023). Increased Expression of HPV-E7 Oncoprotein Correlates with a Reduced Level of pRb Proteins via High Viral Load in Cervical Cancer. Sci. Rep..

[33] Rashid N.N., Yusof R., Watson R.J. (2013). Disruption of Pocket Protein Dream Complexes by E7 Proteins of Different Types of Human Papillomaviruses. Acta Virol..

[34] Janiszewska J., Kostrzewska-Poczekaj M., Wierzbicka M., Brenner J.C., Giefing M. (2024). HPV-Driven Oncogenesis-Much More than the E6 and E7 Oncoproteins. J. Appl. Genet..

[35] Zheng Z.-M., Wang X. (2011). Regulation of Cellular miRNA Expression by Human Papillomaviruses. Biochim. Biophys. Acta BBA-Gene Regul. Mech..

[36] Yim E.-K., Park J.-S. (2005). The Role of HPV E6 and E7 Oncoproteins in HPV-Associated Cervical Carcinogenesis. Cancer Res. Treat..

[37] Chiantore M.V., Mangino G., Iuliano M., Zangrillo M.S., De Lillis I., Vaccari G., Accardi R., Tommasino M., Columba Cabezas S., Federico M. (2016). Human Papillomavirus E6 and E7 Oncoproteins Affect the Expression of Cancer-Related microRNAs: Additional Evidence in HPV-Induced Tumorigenesis. J. Cancer Res. Clin. Oncol..

[38] Ali Syeda Z., Langden S.S.S., Munkhzul C., Lee M., Song S.J. (2020). Regulatory Mechanism of MicroRNA Expression in Cancer. Int. J. Mol. Sci..

[39] Pérez-Mora S., Ocampo-López J., Gómez-García M.D.C., Pérez-Ishiwara D.G. (2023). BFNB Enhances Hair Growth in C57BL/6 Mice through the Induction of EGF and FGF7 Factors and the PI3K-AKT-β-Catenin Pathway. Int. J. Mol. Sci..

[40] Martínez-Cuazitl A., Gómez-García M.D.C., Pérez-Mora S., Rojas-López M., Delgado-Macuil R.J., Ocampo-López J., Vázquez-Zapién G.J., Mata-Miranda M.M., Pérez-Ishiwara D.G. (2023). Polyphenolic Compounds Nanostructurated with Gold Nanoparticles Enhance Wound Repair. Int. J. Mol. Sci..

[41] Zandnia F., Doosti A., Mokhtari-Farsani A., Kardi M.T., Movafagh A. (2016). Application of Multiplex PCR for Rapid and Sensitive Detection of Human Papillomaviruses in Cervical Cancer. Pak. J. Med. Sci..

[42] Williamson A., Rybicki E.P. (1991). Detection of Genital Human Papillomaviruses by Polymerase Chain Reaction Amplification with Degenerate Nested Primers. J. Med. Virol..

[43] Evans M.F., Adamson C.S., Simmons-Arnold L., Cooper K. (2005). Touchdown General Primer (GP5+/GP6+) PCR and Optimized Sample DNA Concentration Support the Sensitive Detection of Human Papillomavirus. BMC Clin. Pathol..

[44] Ocadiz-Delgado R., Castañeda-Saucedo E., Indra A.K., Hernandez-Pando R., Flores-Guizar P., Cruz-Colin J.L., Recillas-Targa F., Perez-Ishiwara G., Covarrubias L., Gariglio P. (2012). RXRα Deletion and E6E7 Oncogene Expression Are Sufficient to Induce Cervical Malignant Lesions in Vivo. Cancer Lett..

[45] Fujinaga Y., Shimada M., Okazawa K., Fukushima M., Kato I., Fujinaga K. (1991). Simultaneous detection and typing of genital human papillomavirus DNA using the polymerase chain reaction. J. Gen. Virol..

[46] Ocadiz-Delgado R., Lizcano-Meneses S., Trejo-Vazquez J., Conde-Perezprina J., Garrido-Palmas F., Alvarez-Rios E., García-Villa E., Ruiz G., Illades-Aguiar B., Leyva-Vázquez M.A. (2021). Circulating miR-15b, miR-34a and miR-218 as Promising Novel Early Low-invasive Biomarkers of Cervical Carcinogenesis. APMIS.

[47] Livak K.J., Schmittgen T.D. (2001). Analysis of Relative Gene Expression Data Using Real-Time Quantitative PCR and the 2−ΔΔCT Method. Methods.

[48] Jackson B.L., Grabowska A., Ratan H.L. (2014). MicroRNA in Prostate Cancer: Functional Importance and Potential as Circulating Biomarkers. BMC Cancer.

[49] Lo U.-G., Yang D., Hsieh J.-T. (2013). The Role of microRNAs in Prostate Cancer Progression. Transl. Androl. Urol..

[50] Nadiminty N., Tummala R., Lou W., Zhu Y., Zhang J., Chen X., eVere White R.W., Kung H.-J., Evans C.P., Gao A.C. (2012). MicroRNA Let-7c Suppresses Androgen Receptor Expression and Activity via Regulation of Myc Expression in Prostate Cancer Cells. J. Biol. Chem..

[51] Mulholland E.J., Green W.P., Buckley N.E., McCarthy H.O. (2019). Exploring the Potential of MicroRNA Let-7c as a Therapeutic for Prostate Cancer. Mol. Ther. Nucleic Acids.

[52] Reis S.T., Timoszczuk L.S., Pontes-Junior J., Viana N., Silva I.A., Dip N., Srougi M., Leite K.R.M. (2013). The Role of Micro RNAs Let7c, 100 and 218 Expression and Their Target RAS, C-MYC, BUB1, RB, SMARCA5, LAMB3 and Ki-67 in Prostate Cancer. Clin. Sao Paulo Braz..

[53] Abdelaal A.M., Sohal I.S., Iyer S.G., Sudarshan K., Orellana E.A., Ozcan K.E., dos Santos A.P., Low P.S., Kasinski A.L. (2024). Selective Targeting of Chemically Modified miR-34a to Prostate Cancer Using a Small Molecule Ligand and an Endosomal Escape Agent. Mol. Ther. Nucleic Acids.

[54] Li W., Liu X., Dougherty E.M., Tang D.G. (2022). MicroRNA-34a, Prostate Cancer Stem Cells, and Therapeutic Development. Cancers.

[55] Liu C., Kelnar K., Liu B., Chen X., Calhoun-Davis T., Li H., Patrawala L., Yan H., Jeter C., Honorio S. (2011). Identification of miR-34a as a Potent Inhibitor of Prostate Cancer Progenitor Cells and Metastasis by Directly Repressing CD44. Nat. Med..

[56] Singh V.K., Rajak N., Singh Y., Singh A.K., Giri R., Garg N. (2024). Role of MicroRNA-21 in Prostate Cancer Progression and Metastasis: Molecular Mechanisms to Therapeutic Targets. Ann. Surg. Oncol..

[57] Gunawan R., Astuti I., Danarto H.R. (2023). miRNA-21 as High Potential Prostate Cancer Biomarker in Prostate Cancer Patients in Indonesia. Asian Pac. J. Cancer Prev..

[58] Hua Y., Liang C., Miao C., Wang S., Su S., Shao P., Liu B., Bao M., Zhu J., Xu A. (2018). MicroRNA-126 Inhibits Proliferation and Metastasis in Prostate Cancer via Regulation of ADAM9. Oncol. Lett..

[59] Jalil A.T., Abdulhadi M.A., Al-Ameer L.R., Abbas H.A., Merza M.S., Zabibah R.S., Fadhil A.A. (2023). The Emerging Role of microRNA-126 as a Potential Therapeutic Target in Cancer: A Comprehensive Review. Pathol. Res. Pract..

[60] Sun X., Liu Z., Yang Z., Xiao L., Wang F., He Y., Su P., Wang J., Jing B. (2013). Association of microRNA-126 Expression with Clinicopathological Features and the Risk of Biochemical Recurrence in Prostate Cancer Patients Undergoing Radical Prostatectomy. Diagn. Pathol..

[61] Hsu T.-I., Hsu C.-H., Lee K.-H., Lin J.-T., Chen C.-S., Chang K.-C., Su C.-Y., Hsiao M., Lu P.-J. (2014). MicroRNA-18a Is Elevated in Prostate Cancer and Promotes Tumorigenesis through Suppressing STK4 in Vitro and in Vivo. Oncogenesis.

[62] Santos S.A.A., Portela L.M.F., Camargo A.C.L., Constantino F.B., Colombelli K.T., Fioretto M.N., Mattos R., de Almeida Fantinatti B.E., Denti M.A., Piazza S. (2022). miR-18a-5p Is Involved in the Developmental Origin of Prostate Cancer in Maternally Malnourished Offspring Rats: A DOHaD Approach. Int. J. Mol. Sci..

[63] Shen K., Cao Z., Zhu R., You L., Zhang T. (2019). The Dual Functional Role of MicroRNA-18a (miR-18a) in Cancer Development. Clin. Transl. Med..

[64] Kiener M., Chen L., Krebs M., Grosjean J., Klima I., Kalogirou C., Riedmiller H., Kneitz B., Thalmann G.N., Snaar-Jagalska E. (2019). miR-221-5p Regulates Proliferation and Migration in Human Prostate Cancer Cells and Reduces Tumor Growth in Vivo. BMC Cancer.

[65] Baruah M.M., Sharma N. (2021). miR-221 Regulates Proliferation, Invasion, Apoptosis and Progression of Prostate Cancer Cells by Modulating E-Cadherin/Wnt/β Catenin Axis. Adv. Cancer Biol.-Metastasis.

[66] Goto Y., Kojima S., Nishikawa R., Kurozumi A., Kato M., Enokida H., Matsushita R., Yamazaki K., Ishida Y., Nakagawa M. (2015). MicroRNA Expression Signature of Castration-Resistant Prostate Cancer: The microRNA-221/222 Cluster Functions as a Tumour Suppressor and Disease Progression Marker. Br. J. Cancer.

[67] Xu W.-X., Liu Z., Deng F., Wang D.-D., Li X.-W., Tian T., Zhang J., Tang J.-H. (2019). MiR-145: A Potential Biomarker of Cancer Migration and Invasion. Am. J. Transl. Res..

[68] Manvati S., Mangalhara K.C., Kalaiarasan P., Chopra R., Agarwal G., Kumar R., Saini S.K., Kaushik M., Arora A., Kumari U. (2019). miR-145 Supports Cancer Cell Survival and Shows Association with DDR Genes, Methylation Pattern, and Epithelial to Mesenchymal Transition. Cancer Cell Int..

[69] Zeinali T., Mansoori B., Mohammadi A., Baradaran B. (2019). Regulatory Mechanisms of miR-145 Expression and the Importance of Its Function in Cancer Metastasis. Biomed. Pharmacother..

[70] Lu J., Mu X., Yin Q., Hu K. (2019). miR-106a Contributes to Prostate Carcinoma Progression through PTEN. Oncol. Lett..

[71] Coman R., Schitcu V., Budisan L., Raduly L., Braicu C., Petrut B., Coman I., Berindan-Neagoe I., Al Hajjar N. (2024). Evaluation of miR-148a-3p and miR-106a-5p as Biomarkers for Prostate Cancer: Pilot Study. Genes.

[72] Garofalo M., Quintavalle C., Romano G., Croce C.M., Condorelli G. (2012). miR221/222 in Cancer: Their Role in Tumor Progression and Response to Therapy. Curr. Mol. Med..

[73] Song Q., An Q., Niu B., Lu X., Zhang N., Cao X. (2019). Role of miR-221/222 in Tumor Development and the Underlying Mechanism. J. Oncol..

[74] Wang D., Sang Y., Sun T., Kong P., Zhang L., Dai Y., Cao Y., Tao Z., Liu W. (2021). Emerging Roles and Mechanisms of microRNA-222-3p in Human Cancer (Review). Int. J. Oncol..

[75] Boulet G., Horvath C., Broeck D.V., Sahebali S., Bogers J. (2007). Human Papillomavirus: E6 and E7 Oncogenes. Int. J. Biochem. Cell Biol..

[76] Swan D.C., Tucker R.A., Tortolero-Luna G., Mitchell M.F., Wideroff L., Unger E.R., Nisenbaum R.A., Reeves W.C., Icenogle J.P. (1999). Human Papillomavirus (HPV) DNA Copy Number Is Dependent on Grade of Cervical Disease and HPV Type. J. Clin. Microbiol..

[77] Jensen J.E., Becker G.L., Jackson J.B., Rysavy M.B. (2024). Human Papillomavirus and Associated Cancers: A Review. Viruses.

[78] Senapati R., Nayak B., Kar S.K., Dwibedi B. (2017). HPV Genotypes Co-Infections Associated with Cervical Carcinoma: Special Focus on Phylogenetically Related and Non-Vaccine Targeted Genotypes. PLoS ONE.

[79] Okadome M., Saito T., Tanaka H., Nogawa T., Furuta R., Watanabe K., Kita T., Yamamoto K., Mikami M., Takizawa K. (2014). Potential Impact of Combined High- and Low-risk Human Papillomavirus Infection on the Progression of Cervical Intraepithelial Neoplasia 2. J. Obstet. Gynaecol. Res..

[80] Campos R.G., Malacara Rosas A., Gutiérrez Santillán E., Delgado Gutiérrez M., Torres Orozco R.E., García Martínez E.D., Torres Bernal L.F., Rosas Cabral A. (2019). Unusual Prevalence of High-Risk Genotypes of Human Papillomavirus in a Group of Women with Neoplastic Lesions and Cervical Cancer from Central Mexico. PLoS ONE.

[81] De Brot L., Pellegrini B., Moretti S.T., Carraro D.M., Soares F.A., Rocha R.M., Baiocchi G., Da Cunha I.W., De Andrade V.P. (2017). Infections with Multiple High-risk HPV Types Are Associated with High-grade and Persistent Low-grade Intraepithelial Lesions of the Cervix. Cancer Cytopathol..

[82] Luo Q., Zeng X., Luo H., Pan L., Huang Y., Zhang H., Han N. (2023). Epidemiologic Characteristics of High-Risk HPV and the Correlation between Multiple Infections and Cervical Lesions. BMC Infect. Dis..

[83] Wu Y., Godoy A., Azzouni F., Wilton J.H., Ip C., Mohler J.L. (2013). Prostate Cancer Cells Differ in Testosterone Accumulation, Dihydrotestosterone Conversion, and Androgen Receptor Signaling Response to Steroid 5α-Reductase Inhibitors. The Prostate.

[84] García Lozano T., García García E., González Monsalve J.A., Illueca Ballester C., Aznar Oroval E., San Juan Gadea M.C., Navarro Gallego M.T., Almenar Medina S. (2015). Análisis de las coinfecciones mixtas por el virus del papiloma humano (VPH) de alto y bajo riesgo en lesiones de significado incierto. Clínica E Investig. En Ginecol. Obstet..

[85] Mekhail S.M., Yousef P.G., Jackinsky S.W., Pasic M., Yousef G.M. (2014). miRNA in Prostate Cancer: New Prospects for Old Challenges. EJIFCC.

[86] Zhang R., Su J., Xue S.-L., Yang H., Ju L.-L., Ji Y., Wu K.-H., Zhang Y.-W., Zhang Y.-X., Hu J.-F. (2016). HPV E6/P53 Mediated down-Regulation of miR-34a Inhibits Warburg Effect through Targeting LDHA in Cervical Cancer. Am. J. Cancer Res..

[87] Khatami A., Nahand J.S., Kiani S.J., Khoshmirsafa M., Moghoofei M., Khanaliha K., Tavakoli A., Emtiazi N., Bokharaei-Salim F. (2022). Human Papilloma Virus (HPV) and Prostate Cancer (PCa): The Potential Role of HPV Gene Expression and Selected Cellular MiRNAs in PCa Development. Microb. Pathog..

[88] Lajer C.B., Garnæs E., Friis-Hansen L., Norrild B., Therkildsen M.H., Glud M., Rossing M., Lajer H., Svane D., Skotte L. (2012). The Role of miRNAs in Human Papilloma Virus (HPV)-Associated Cancers: Bridging between HPV-Related Head and Neck Cancer and Cervical Cancer. Br. J. Cancer.

[89] Wagner S., Ngezahayo A., Murua Escobar H., Nolte I. (2014). Role of miRNA *Let-7* and Its Major Targets in Prostate Cancer. BioMed Res. Int..

[90] Ma Y., Shen N., Wicha M.S., Luo M. (2021). The Roles of the Let-7 Family of MicroRNAs in the Regulation of Cancer Stemness. Cells.

[91] Zhang W.-T., Zhang G.-X., Gao S.-S. (2021). The Potential Diagnostic Accuracy of Let-7 Family for Cancer: A Meta-Analysis. Technol. Cancer Res. Treat..

[92] Dong Q., Meng P., Wang T., Qin W., Qin W., Wang F., Yuan J., Chen Z., Yang A., Wang H. (2010). MicroRNA Let-7a Inhibits Proliferation of Human Prostate Cancer Cells In Vitro and In Vivo by Targeting E2F2 and CCND2. PLoS ONE.

[93] Alwhaibi A., Parvathagiri V., Verma A., Artham S., Adil M.S., Somanath P.R. (2022). Regulation of Let-7a-5p and miR-199a-5p Expression by Akt1 Modulates Prostate Cancer Epithelial-to-Mesenchymal Transition via the Transforming Growth Factor-β Pathway. Cancers.

[94] Li B., Guo X., Li N., Chen Q., Shen J., Huang X., Huang G., Wang F. (2020). WNT1, a Target of miR-34a, Promotes Cervical Squamous Cell Carcinoma Proliferation and Invasion by Induction of an E-P Cadherin Switch via the WNT/β-Catenin Pathway. Cell. Oncol. Dordr..

[95] Feng Y.-H., Tsao C.-J. (2016). Emerging Role of microRNA-21 in Cancer. Biomed. Rep..

[96] Stafford M.Y.C., Willoughby C.E., Walsh C.P., McKenna D.J. (2022). Prognostic Value of miR-21 for Prostate Cancer: A Systematic Review and Meta-Analysis. Biosci. Rep..

[97] Chen Q., Chen S., Zhao J., Zhou Y., Xu L. (2021). MicroRNA-126: A New and Promising Player in Lung Cancer. Oncol. Lett..

[98] Zhou Y., Feng X., Liu Y., Ye S., Wang H., Tan W., Tian T., Qiu Y., Luo H. (2013). Down-Regulation of miR-126 Is Associated with Colorectal Cancer Cells Proliferation, Migration and Invasion by Targeting IRS-1 via the AKT and ERK1/2 Signaling Pathways. PLoS ONE.

[99] Miao Y., Lu J., Fan B., Sun L. (2020). MicroRNA-126-5p Inhibits the Migration of Breast Cancer Cells by Directly Targeting CNOT7. Technol. Cancer Res. Treat..

[100] Lu H., Gu X. (1019). MicroRNA-221 Inhibits Human Papillomavirus 16 E1-E2 Mediated DNA Replication through Activating SOCS1/Type I IFN Signaling Pathway. Int. J. Clin. Exp. Pathol..

[101] Di Martino M.T., Arbitrio M., Caracciolo D., Cordua A., Cuomo O., Grillone K., Riillo C., Caridà G., Scionti F., Labanca C. (2022). miR-221/222 as Biomarkers and Targets for Therapeutic Intervention on Cancer and Other Diseases: A Systematic Review. Mol. Ther. Nucleic Acids.

[102] Ye T., Zhong L., Ye X., Liu J., Li L., Yi H. (2021). miR-221-3p and miR-222-3p Regulate the SOCS3/STAT3 Signaling Pathway to Downregulate the Expression of NIS and Reduce Radiosensitivity in Thyroid Cancer. Exp. Ther. Med..

[103] Mishra S., Deng J.J., Gowda P.S., Rao M.K., Lin C.-L., Chen C.L., Huang T., Sun L.-Z. (2014). Androgen Receptor and MicroRNA-21 Axis down-Regulates Transforming Growth Factor Beta Receptor II (TGFBR2) Expression in Prostate Cancer. Oncogene.

[104] Kneitz B., Krebs M., Kalogirou C., Schubert M., Joniau S., Van Poppel H., Lerut E., Kneitz S., Scholz C.J., Ströbel P. (2014). Survival in Patients with High-Risk Prostate Cancer Is Predicted by miR-221, Which Regulates Proliferation, Apoptosis, and Invasion of Prostate Cancer Cells by Inhibiting IRF2 and SOCS3. Cancer Res..

[105] Cui S.-Y., Wang R., Chen L.-B. (2014). MicroRNA-145: A Potent Tumour Suppressor That Regulates Multiple Cellular Pathways. J. Cell. Mol. Med..

[106] Hart M., Wach S., Nolte E., Szczyrba J., Menon R., Taubert H., Hartmann A., Stoehr R., Wieland W., Grässer F.A. (2013). The Proto-oncogene ERG Is a Target of Micro RNA
*miR-145* in Prostate Cancer. FEBS J..

[107] Tahamtan A., Teymoori-Rad M., Nakstad B., Salimi V. (2018). Anti-Inflammatory MicroRNAs and Their Potential for Inflammatory Diseases Treatment. Front. Immunol..

[108] Gunasekharan V., Laimins L.A. (2013). Human Papillomaviruses Modulate MicroRNA 145 Expression to Directly Control Genome Amplification. J. Virol..

[109] Miller D.L., Davis J.W., Taylor K.H., Johnson J., Shi Z., Williams R., Atasoy U., Lewis J.S., Stack M.S. (2015). Identification of a Human Papillomavirus–Associated Oncogenic miRNA Panel in Human Oropharyngeal Squamous Cell Carcinoma Validated by Bioinformatics Analysis of The Cancer Genome Atlas. Am. J. Pathol..

