# Supplementary material for: miRNAs Dysregulated in Human Papillomavirus-Associated Benign Prostatic Lesions and Prostate Cancer
Source: Cancers (Basel). 2024 Dec 25;17(1):26. doi: 10.3390/cancers17010026 (PMC11718816; doi:10.3390/cancers17010026)
Supplement: Supplementary file 1 [file cancers-17-00026-s001.zip › cancers-3342714-supplementary.pdf]

Supplementary Table S1. Interactome analysis of miRNAs implicated in prostate cancer.

	miRNAs
Interactomic analysis by miRNet	hsa-let-7a-1, hsa-let-7a-2, hsa-let-7a-3, hsa-let-7b, **hsa-let-7c**, hsa-let-7d, hsa-let-7e, hsa-let-7f-1, hsa-let-7f-2, hsa-mir-15a, hsa-mir-16-1, hsa-mir-17, **hsa-mir-18a**, hsa-mir-19a, hsa-mir-19b-1, hsa-mir-19b-2, hsa-mir-20a, **hsa-mir-21**, hsa-mir-22, hsa-mir-23a, hsa-mir-24-1, hsa-mir-24-2, hsa-mir-25, hsa-mir-26a-1, hsa-mir-26b, hsa-mir-27a, hsa-mir-28, hsa-mir-29a, hsa-mir-30a, hsa-mir-31, hsa-mir-32, hsa-mir-33a, hsa-mir-92a-1, hsa-mir-92a-2, hsa-mir-93, hsa-mir-95, hsa-mir-96, hsa-mir-98, hsa-mir-99a, hsa-mir-100, hsa-mir-101-1, hsa-mir-29b-1, hsa-mir-29b-2, hsa-mir-103-2, hsa-mir-103a-2, hsa-mir-103-1, hsa-mir-103a-1, hsa-mir-105-1, hsa-mir-105-2, hsa-**mir-106a**, hsa-mir-16-2, hsa-mir-196a-1, hsa-mir-197, hsa-mir-199a-1, hsa-mir-208a, hsa-mir-148a, hsa-mir-30c-2, hsa-mir-30d, hsa-mir-139, hsa-mir-7-1, hsa-mir-7-2, hsa-mir-7-3, hsa-mir-10a, hsa-mir-10b, **hsa-mir-34a**, hsa-mir-181a-2, hsa-mir-181b-1, hsa-mir-181c, hsa-mir-182, hsa-mir-183, hsa-mir-187, hsa-mir-196a-2, hsa-mir-199a-2, hsa-mir-199b, hsa-mir-203, hsa-mir-203a, hsa-mir-204, hsa-mir-205, hsa-mir-210, hsa-mir-211, hsa-mir-212, hsa-mir-181a-1, hsa-mir-214, hsa-mir-215, hsa-mir-216a, hsa-mir-218-1, hsa-mir-218-2, **hsa-mir-221**, **hsa-mir-222**, hsa-mir-223, hsa-mir-224, hsa-mir-200b, hsa-let-7g, hsa-let-7i, hsa-mir-1-2, hsa-mir-15b, hsa-mir-23b, hsa-mir-27b, hsa-mir-30b, hsa-mir-122, hsa-mir-124-1, hsa-mir-124-2, hsa-mir-124-3, hsa-mir-125b-1, hsa-mir-128-1, hsa-mir-130a, hsa-mir-132, hsa-mir-133a-1, hsa-mir-133a-2, hsa-mir-135a-1, hsa-mir-135a-2, hsa-mir-138-2, hsa-mir-141, hsa-mir-142, **hsa-mir-143**, hsa-mir-144, **hsa-mir-145**, hsa-mir-152, hsa-mir-153-1, hsa-mir-153-2, hsa-mir-191, hsa-mir-9-1, hsa-mir-9-2, hsa-mir-9-3, hsa-mir-125a, hsa-mir-125b-2, **hsa-mir-126**, hsa-mir-127, hsa-mir-129-2, hsa-mir-134, hsa-mir-136, hsa-mir-138-1, hsa-mir-146a, hsa-mir-149, hsa-mir-150, hsa-mir-154, hsa-mir-185, hsa-mir-186, hsa-mir-188, hsa-mir-193a, hsa-mir-194-1, hsa-mir-195, hsa-mir-200c, hsa-mir-1-1, hsa-mir-155, hsa-mir-181b-2, hsa-mir-128-2, hsa-mir-194-2, hsa-mir-106b, hsa-mir-29c, hsa-mir-30c-1, hsa-mir-200a, hsa-mir-302a, hsa-mir-101-2, hsa-mir-34b, hsa-mir-299, hsa-mir-301a, hsa-mir-99b, hsa-mir-296, hsa-mir-130b, hsa-mir-30e, hsa-mir-26a-2, hsa-mir-361, hsa-mir-365-2, hsa-mir-365b, hsa-mir-302b, hsa-mir-302c, hsa-mir-302d, hsa-mir-367, hsa-mir-376c, hsa-mir-369, hsa-mir-370, hsa-mir-372, hsa-mir-373, hsa-mir-374a, hsa-mir-376a-1, hsa-mir-377, hsa-mir-379, hsa-mir-380, hsa-mir-381, hsa-mir-382, hsa-mir-383, hsa-mir-340, hsa-mir-330, hsa-mir-328, hsa-mir-342, hsa-mir-337, hsa-mir-151, hsa-mir-151a, hsa-mir-135b, hsa-mir-148b, hsa-mir-331, hsa-mir-324, hsa-mir-338, hsa-mir-339, hsa-mir-335, hsa-mir-345, hsa-mir-196b, hsa-mir-423, hsa-mir-424, hsa-mir-425, hsa-mir-491, hsa-mir-146b, hsa-mir-202, hsa-mir-497, hsa-mir-503, hsa-mir-513a-1, hsa-let-7a, hsa-let-7a-5p, hsa-let-7f, hsa-let-7f-5p, hsa-mir-16, hsa-mir-16-5p, hsa-mir-17-5p, hsa-mir-17*, hsa-mir-17-3p, hsa-mir-19b, hsa-mir-19b-3p, hsa-mir-24, hsa-mir-24-3p, hsa-mir-26a, hsa-mir-26a-5p, hsa-mir-30a-5p, hsa-mir-92a, hsa-mir-92a-3p, hsa-mir-101, hsa-mir-101-3p, hsa-mir-29b, hsa-mir-29b-3p, hsa-mir-103, hsa-mir-103a, hsa-mir-103a-3p, hsa-mir-107, hsa-mir-198, hsa-mir-199a-5p, hsa-mir-199a-3p, hsa-mir-30c, hsa-mir-30c-5p, hsa-mir-147, hsa-mir-147a, hsa-mir-181b, hsa-mir-181b-5p, hsa-mir-182*, hsa-mir-182-3p, hsa-mir-217, hsa-mir-218, hsa-mir-218-5p, hsa-mir-137, hsa-mir-126*, hsa-mir-126-5p, hsa-mir-184, hsa-mir-190, hsa-mir-190a, hsa-mir-190a-5p, hsa-mir-206, hsa-mir-320a, hsa-mir-302c*, hsa-mir-302c-5p, hsa-mir-371-3p, hsa-mir-371a-3p, hsa-mir-373*, hsa-mir-373-5p, hsa-mir-375, hsa-mir-378*, hsa-mir-378a-5p, hsa-mir-323-3p, hsa-mir-323a-3p, hsa-mir-326, hsa-mir-133b, hsa-mir-325, hsa-mir-346, hsa-mir-422a, hsa-mir-449a, hsa-mir-498, hsa-mir-521, hsa-mir-513a-5p, hsa-mir-663, hsa-mir-663a, hsa-mir-15a*, hsa-mir-15a-3p, hsa-mir-219-1-3p, hsa-mir-219a-1-3p, hsa-mir-219-2-3p, hsa-mir-219a-2-3p.

The miRNAs highlighted in red represent those whose expression we evaluated across the different experimental groups in our study.
